# Performance of Pristine *versus* Magnetized Orange Peels Biochar Adapted to Adsorptive Removal of Daunorubicin: Eco-Structuring, Kinetics and Equilibrium Studies

**DOI:** 10.3390/nano13091444

**Published:** 2023-04-23

**Authors:** Ahmed S. El-Shafie, Farahnaz G. Barah, Maha Abouseada, Marwa El-Azazy

**Affiliations:** Department of Chemistry and Earth Sciences, College of Arts and Sciences, Qatar University, Doha 2713, Qatar

**Keywords:** orange peels biochar, magnetic biochar, daunorubicin, Plackett–Burman design, desirability function

## Abstract

Drugs and pharmaceuticals are an emergent class of aquatic contaminants. The existence of these pollutants in aquatic bodies is currently raising escalating concerns because of their negative impact on the ecosystem. This study investigated the efficacy of two sorbents derived from orange peels (OP) biochar (OPBC) for the removal of the antineoplastic drug daunorubicin (DNB) from pharmaceutical wastewater. The adsorbents included pristine (OPBC) and magnetite (Fe_3_O_4_)-impregnated (MAG-OPBC) biochars. Waste-derived materials offer a sustainable and cost-effective solution to wastewater bioremediation. The results showed that impregnation with Fe_3_O_4_ altered the crystallization degree and increased the surface area from 6.99 m^2^/g in OPBC to 60.76 m^2^/g in the case of MAG-OPBC. Placket–Burman Design (PBD) was employed to conduct batch adsorption experiments. The removal efficiency of MAG-OPBC (98.51%) was higher compared to OPBC (86.46%). DNB adsorption onto OPBC followed the D–R isotherm, compared to the Langmuir isotherm in the case of MAG-OPBC. The maximum adsorption capacity (*q_max_*) was 172.43 mg/g for MAG-OPBC and 83.75 mg/g for OPBC. The adsorption kinetics for both sorbents fitted well with the pseudo-second-order (PSO) model. The results indicate that MAG-OPBC is a promising adsorbent for treating pharmaceutical wastewater.

## 1. Introduction

The past few decades have seen a growing use of antineoplastics for chemotherapy. Many of the cytostatic drugs, though being used for abatement of cancer, have been classified as CMR chemicals, i.e., carcinogenic, mutagenic, and reprotoxic. The escalating consumption and the associated risks are prompting inevitable concerns related to their occupational exposure and the consequent ecotoxicological threats [1,2]. By and large, the rates of consumption of cytostatic drugs are relatively low compared to the other pharmaceutically active compounds. Their existence in the water cycle, hence, seems questionable. Parallel to that, the environmental risk assessment process is usually executed for newly permitted pharmaceuticals, therefore, the majority of cytostatic drugs might not be exposed to such an assessment. With these considerations, the investigations targeting the presence and the removal of cytostatic drugs are limited [2,3].

**Scheme 1:**
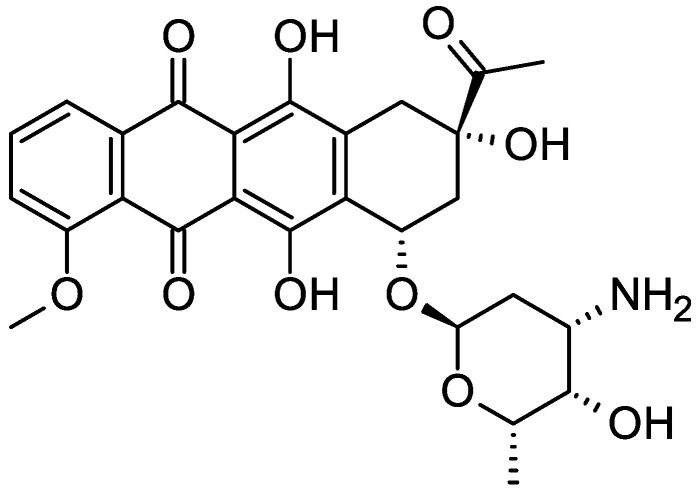
Chemical structure of daunorubicin (DNB), molar mass (DNB hydrochloride) = 527.52 g/mol, pK_a1_ = 7.48, pK_a2_ = 9.68 [7].

Daunorubicin (DNB), also known as daunomycin, Scheme 1, is a cytostatic antibiotic that belongs to the family of anthracyclines. DNB is commonly used in the treatment of different types of acute leukemia and as a combination therapy in the treatment of lymphoma, breast, and bladder cancers [4]. Being widely used, DNB is on the World Health Organization’s List of Essential Medicines [5]. As per the International Agency on the Research of Cancer (IARC), DNB is classified as category 2B carcinogenic (possibly carcinogenic) [6]. Compared to the other family members, higher percentage, around 13–15% of DNB, is excreted unmetabolized in urine within 24 h. DNB was detected in the range of 260–1350 ng/L in oncological wards wastewaters [1]. The detection of DNB in such high concentrations raises concerns about the efficiency of the wastewater treatment approaches currently pursued.

Literature surveying shows that not much attention has been paid to the removal of antineoplastics from contaminated waters, especially DNB. Therefore, the ecological impact of DNB and its fate remain poorly comprehended. Table 1 shows a glance at literature efforts made to remove antineoplastics from different water matrices. Removal strategies included adsorption, nanofiltration, photocatalysis, membrane bioreactors, and Fenton/ozone oxidation processes [7,8,9,10,11,12,13,14].

Adsorption of pharmaceuticals onto biochars, especially those generated via the pyrolysis of agro-waste biomasses, has been the subject of several investigations [15,16,17,18,19,20,21]. However, much fewer attempts have been made to remove the cytostatic drugs via sorption onto biochars. Within the continuous rivalries for developing green solutions for water remediation and under the umbrella of circular economy, recycling agro-waste materials into value-added products is a widely used approach. On the one hand, agro-waste materials are available at almost no cost and could represent a burden on the environment if not properly recycled [22,23,24]. On the other hand, biochars obtained via pyrolysis of the lignocellulosic materials are greatly privileged with their high surface area, pore size and volume, existence of various functionalities, and liability for functionalization and regeneration, making them excellent candidates for scavenging of contaminants [25,26,27,28,29,30].

Within these premises, orange peels (*Citrus sinensis*) are ubiquitously and copiously available as a byproduct of the juice processing industry. The world production of oranges amounted to almost half of the global production of citrus fruits, 124 × 10^6^ tons, in 2016, as per the UN Food and Agriculture Organization report [31]. According to this report, the total imports of Qatar from citrus totaled 46.4 × 10^3^ tons in 2016, with oranges representing almost half. Recycling waste peels is, therefore, a sustainable solution, especially in the major producing countries.

Parallel with the sustainability offered by the valorization of agro-wastes, functionalization of the biochar with magnetite (Fe_3_O_4_) nanoparticles is a plus. Magnetic nanosorbents possess high surface area and could be used on a large scale to treat large volumes of water thanks to the feasibility of their separation using a low-strength external magnetic field [32,33,34,35,36]. In the current investigation, biochar of orange peels (OPBC) and the magnetite-loaded biochar (MAG-OPBC) will be synthesized and exploited for the removal of DNB from synthetic wastewater.

Application of both sorbents in treating contaminated samples will be controlled by implementing a multivariate-based stratagem. In this itinerary, and in alignment with the greenness preservation, Plackett–Burman design (PBD) will be executed as the screening design, followed by an optimization phase using the desirability function [18,37,38,39]. The target is to get the utmost response with the lowest possible usage of resources and hence generation of waste. Four variables that are expected to impact the removal efficiency (%R) of the two sorbents will be assessed. The objective will be set to have the maximum %R as a function of the independent variables: pH, biochar dose (AD), [DNB] and contact time (CT). The novelty of this approach, therefore, stems from being, to the best of authors’ knowledge, the first sustainable and green approach that deals with using the biochar of orange peels as pristine and loaded with magnetite in removing DNB executing a multivariate-based scheme.

## 2. Materials and Methods

### 2.1. Chemicals

In this study, deionized water was taken from a Millipore-Q water system (Burlington, MA, USA), while all reagents used were of analytical grade and used as purchased with no additional purification. Daunorubicin (DNB) was purchased from Biosynth^®^ Carbosynth Ltd. (Compton, Berkshire, UK). Hydrochloric acid, sodium hydroxide, ammonium iron (III) sulfate dodecahydrate (NH_4_Fe(SO_4_)_2_·12H_2_O), and ferrous ammonium sulfate hexahydrate (Fe(NH_4_)_2_(SO_4_)_2_·6H_2_O) were procured from Sigma–Aldrich (St. Louis, MO, USA). Navel orange peels were obtained from a juice store in Doha, Qatar.

### 2.2. Preparation of OPBC

The orange peels underwent a thorough cleaning process, which involved washing five times with distilled water and another five times with deionized water. The peels were then sliced into smaller cuts, roughly 2 × 2 cm, and were allowed to dry out in open air (in the shade) for three days. In the next stage, the peels were further dried in an oven at 80 °C for three successive days until they became brittle. Once entirely dry, the peels were crushed into a fine powder. A clean and dry crucible was used to hold 10 g of the powdered peels, which were then covered up with a cap and disposed of in a furnace. The crucible was sealed and heated at 500 °C for 1 h. Afterwards, the crucible was let to cool to room temperature, and the peels were divided into two portions, labeled as OPBC, and kept in the desiccator for future use.

### 2.3. Preparation of MAG-OPBC

The magnetic biochar known as MAG-OPBC was produced utilizing a co-precipitation technique [40,41]. The process involved mixing a 200 mL aqueous solution of 0.50 M Fe(II) with a 400 mL equimolar solution of Fe(III) and agitating the resulting mixture at 600 rpm and 60 °C for 1 h. Subsequently, 10 g of OPBC was added to the mixture and stirred at 60 °C for 3 h. A 4 M aqueous solution of sodium hydroxide was then introduced to the suspension slowly until the pH was ~12. After 30 min. of gentle stirring at room temperature, the suspension underwent 10 washes with distilled water followed by 5 additional washes with methanol. The MAG-OPBC that was obtained was filtered under vacuum and dried up overnight at 70 °C before being stored in uncontaminated vials for future applications.

### 2.4. Measurment of the Point-of-Zero-Charge (pH_PZC_)

To determine the pH_PZC_ of OPBC and MAG-OPBC, 1.0000 ± 0.0005 g of each adsorbent was placed into seven separate beakers with 50 mL of aqueous sodium chloride solution (0.01 M). The pH of the solution was then adjusted within the studied range of 2.5–10.0 ± 0.2, using diluted solutions of either hydrochloric acid or sodium hydroxide. The samples were mixed for 24 h at 200 rpm using a mechanical shaker before detecting the final pH. pH_PZC_ for both OPBC and MAG-OPBC was then calculated by intersecting the pH_final_ and pH_initial_ curves [42].

### 2.5. Characterization of OPBC and MAG-OPBC

Thermal gravimetric analysis (TGA, PerkinElmer-TGA400, Waltham, MA, USA) was carried out at a temperature range of 30–900 °C at a rate of 10.00 °C/min and in the presence of air. TGA was used to investigate the thermal stability of the prepared adsorbents. Fourier transform infrared spectroscopy (FT-IR, Bruker Alpha, MA, USA) was employed to verify the presence of functional groups on the adsorbent surface. Raman spectroscopy (Thermo Scientific, Waltham, MA, USA) was used to confirm the conversion of peels into carbonaceous material and the formation of magnetite nanoparticles on the surface of the adsorbent. The sample was first ground with potassium bromide to form a thin and homogeneous mixture. The mixture was then compressed into a pellet using a hydraulic press to produce a thin, transparent film, which was used for FT-IR and Raman analyses. To examine the morphology and textural properties of both adsorbents, a scanning electron microscope (SEM, FEI, Quanta 200, Thermo Scientific, Waltham, MA, USA) was utilized. Moreover, the elemental composition of both samples was determined using an energy-dispersive X-ray spectrometer (EDX). A transmission electron microscope (TEM, FEI, TECNAI G2 TEM, TF20) was used to characterize the microstructure of both sorbents. The preparation of the TEM sample for both magnetic and non-magnetic sorbents was done by dispersing the sample in warm distilled water followed by ultrasonic mixing for 30 min. The samples were subsequently mounted on a carbon-coated grid. Porosity and surface area were measured using a Micromeritics ASAP^TM^ 2020 accelerated surface area and porosimetry system. Before N_2_ adsorption-desorption, the samples were degassed, and isotherms collected at 77 K were used to calculate the surface area using the Brunauer–Emmett–Teller (BET) equation. The t-plots and the Barrett–Joyner–Halenda (BJH) equations were utilized to find the pore volume.

### 2.6. Batch Adsorption Experiments

For the batch experimental runs, a stock solution of DNB in water with a concentration of 100 ppm was used. The pH of the water used to suspend OPBC and MAG-OPBC was adjusted to the desired levels by adding 0.1 M solutions of hydrochloric acid or sodium hydroxide, as specified in Table 2. A pH meter from Jenway (Cole-Parmer, Stone, Staffordshire, UK) was utilized to measure the pH, while a UV-Vis spectrophotometer from Agilent (Santa Clara, CA, USA) with matched quartz cells of 10 mm was used to determine the concentrations of DNB before and after it was adsorbed onto the sorbent. The supernatant solution was filtered using a non-sterile nylon Millex syringe filter with a pore size of 0.45 µm.

### 2.7. Plackett–Burman Design Execution

To spot the statistically significant variables for better removal of DNB, a saturated orthogonal statistical screening design, PBD was executed [43,44]. Herein, the design involved 26 experimental runs (encompassing 6 central points, Ct Pt) for 4 independent variables coded as A, B, C, and D, as shown in Table 2. All variables were of the numerical type. All 26 experimental runs were performed in triplicate, and an average value was recorded as the dependent response, %R. Obtained results were further validated versus the predicted values provided by Minitab^®^. Precise optimization of the statistically significant variables was performed in a subsequent phase using the desirability function tool (*d).* The coefficient of determination (R^2^), adjusted-R^2^ (R^2^-adj), and predicted R^2^ (R^2^-pred), as well as the analysis of variance (ANOVA) and the impact of the standardized effects of all variables on DNB removal, were determined using Minitab^®^ 19 software. 

### 2.8. Equilibrium and Kinetics Investigation

Deionized water was used to prepare a range of dilutions for DNB, with concentrations varying from 5–300 ppm. The pH of the solutions was attuned to 9.0 ± 0.2. A mass of 0.1000 ± 0.0005 g of both OPBC and MAG-OPBC was added to each drug solution, and the mixture was shaken for 48 h at 200 rpm using an automatic shaker. The resulting solutions were filtered, and the absorbance was measured at 496 nm.

To evaluate the adsorption kinetics of DNB onto OPBC and MAG-OPC, 200 mL of DNB solution (at a concentration of 100 ppm and a pH of 9.0 ± 0.2) was added to 0.5000 ± 0.0005 g of the adsorbent and stirred continuously at 500 rpm. Aliquots of 10 mL were taken from the reaction mixture at various time intervals over 150 min and filtered. The absorbance of the resulting filtrate was then recorded at 496 nm.

## 3. Results

### 3.1. Characterization and Surface Chemistry

#### 3.1.1. Thermogravimetric Analysis (TGA)

**Figure 1:**
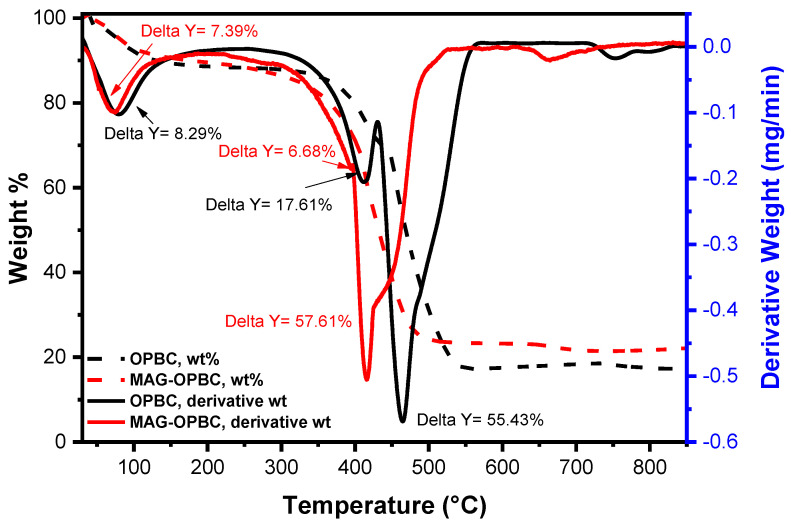
TGA/*dTA* analysis of OPBC and MAG-OPBC.

Figure 1 displays the TGA/*d*TA analysis findings. The results obtained indicate that the OPBC and MAG-OPBC samples exhibited a weight loss of 8.29 and 7.39%, respectively, in the temperature range of 50–200 °C corresponding to the evaporation of free water. Additionally, in the range of 350–600 °C, there was a weight loss for both OPBC and MAG-OPBC samples, 73.04% and 64.29%, respectively. This weight loss could be ascribed to the loss of the organic matter and the carbonization of the polymeric components in both samples. The total weight loss for OPBC was 82.68%, compared to 77.61% in the case of MAG-OPBC. The difference in the weight loss between the two samples could be attributed to the presence of magnetic nanoparticles on the surface of MAG-OPBC, which increased the thermal stability of the nanosorbent.

#### 3.1.2. FT-IR Analysis and Point-of-Zero-Charge (pH_PZC_)

**Figure 2:**
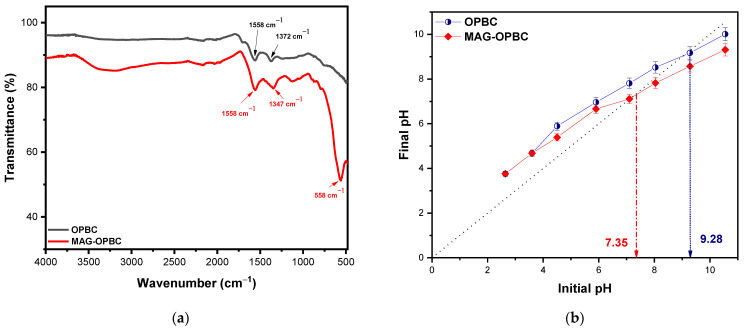
FT−IR spectra of (**a**) OPBC and MAG-OPBC and (**b**) pH_PZC_ of both adsorbents.

FT-IR analysis was carried out to identify the functional groups present on the surface of OPBC and MAG-OPBC adsorbents and hence explore the adsorption mechanism (Figure 2a). The results showed similarities in the spectra of both adsorbents, with some peak shifts observed following the impregnation with Fe_3_O_4_. An absorption band at 1558 cm^−1^ appeared in both samples, corresponding to carboxylic C=O or aromatic C=C stretching vibrations. The peak at 1372 cm^−1^ and 1347 cm^−1^ observed in OPBC, and MAG-OPBC, respectively, was assigned to C-O stretching, and the shift indicates bond formation between Fe_3_O_4_ and the surface of the biochar [45]. The presence of an absorption band at 558 cm^−1^ could be attributed to the Fe-O bond, and it confirms the existence of magnetite on the surface [46,47,48,49,50]. Figure 2b illustrates the point-of-zero-charge for OPBC and MAG-OPBC, which was found to be 9.28 and 7.35, respectively. This implies that if the pH of the solution is higher than pH_PZC_, the surface of OPBC or MAG-OPBC would be negatively charged. This property, together with the pKa value of DNB, is fundamental in deciding upon the adsorption mechanism and hence the efficiency of the studied adsorbent towards the target pollutant.

#### 3.1.3. CHN Analysis

The data of the CHN analysis for both OPBC and MAG-OPBC indicate that the N% increased from 1.83% in the case of OPBC to 2.21% in the case of MAG-OPBC. Besides, the C% decreased from 64.33% in OPBC to 64.21% in MAG-OPBC due to the existence of magnetic nanoparticles on the surface. Similarly, the H% decreased from 2.34% in OPBC to 2.29% in MAG-OPBC due to the loss of hydrogen during the formation of Fe_3_O_4_ on the surface of the nanosorbent.

#### 3.1.4. Raman Analysis 

**Figure 3:**
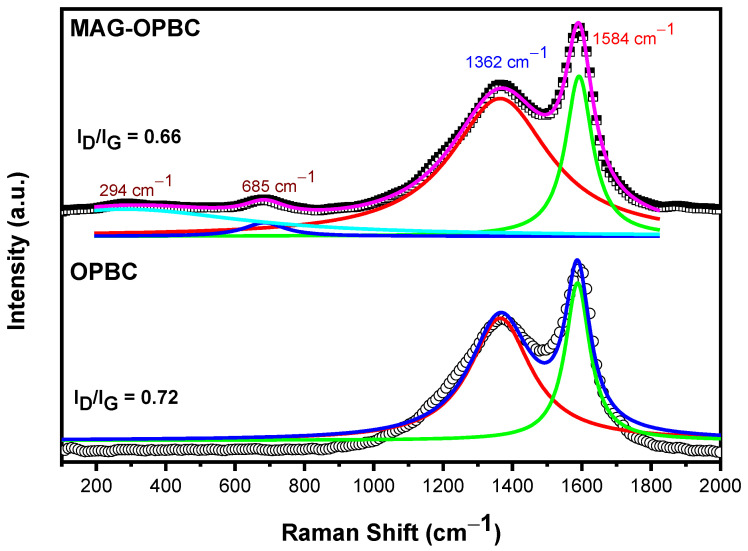
Raman spectra of the as-prepared samples OPBC and MAG-OPBC.

Figure 3 portrays the Raman spectra of the pristine adsorbent, OPBC and their as-prepared sample after impregnation by magnetic nanoparticles (MAG-OPBC). The data obtained in the case of OPBC show two distinct bands linked with carbonous materials and could be observed at 1362 cm^−1^ (D-band) and 1584 cm^−1^ (G-band), which are typically related to carbonaceous materials derived from thermally treated peels. The D-band indicates the carbon lattice attributes, including defects and sizes, while the G-band detects C-C stretching for the *sp*^2^ structure. Additionally, the I_D_:I_G_ intensity ratio for OPBC is 0.72, whereas it is 0.66 for MAG-OPBC. This suggests that the number of defects on the surface of OPBC decreased after impregnation with Fe_3_O_4_. The presence of Fe_3_O_4_ nanoparticles onto OPBC was confirmed by the occurrence of two weak broad peaks in the spectrum of MAG-OPBC located at 294 and 685 cm^−1^, which could be credited to the existence of Fe–O bonds of the magnetic nanoparticles [51,52,53].

#### 3.1.5. BET Analysis

**Figure 4:**
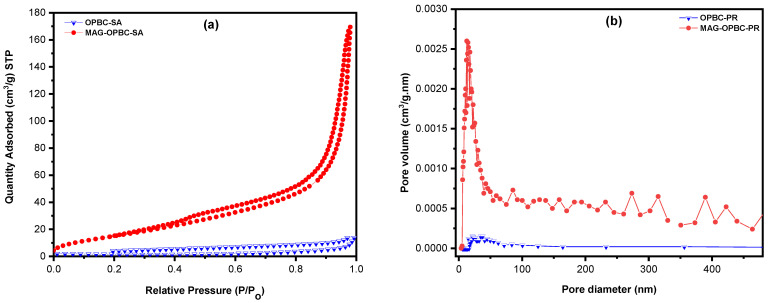
(**a**) Surface area and (**b**) the pore diameter for both adsorbents OPBC, and MAG-OPBC.

The BET equation was employed to calculate the surface area of OPBC and MAG-OPBC, Table S1. Figure 4 presents the N_2_ adsorption-desorption isotherms for both samples. The obtained data revealed that the Langmuir surface area has increased from 6.99 m^2^/g in OPBC to 60.76 m^2^/g for MAG-OPBC, possibly due to the existence of Fe_3_O_4_ nanoparticles on the surface of OPBC, which enhances the surface area and improves the efficacy of DNB removal. Mesopores (2–50 nm) and macropores (>50 nm) were also observed in both samples. The adsorption isotherm for both sorbents was of type IV, indicating monolayer-multilayer adsorption with subsequent capillary condensation. The H3-type hysteresis loop was also observed, which is typical for materials with a broad range of pore diameters and indicates the formation of slit-like pores from loose masses of plate-like particles [54].

#### 3.1.6. Morphological and Microstructural Features of OPBC and MAG-OPBC

**Figure 5:**
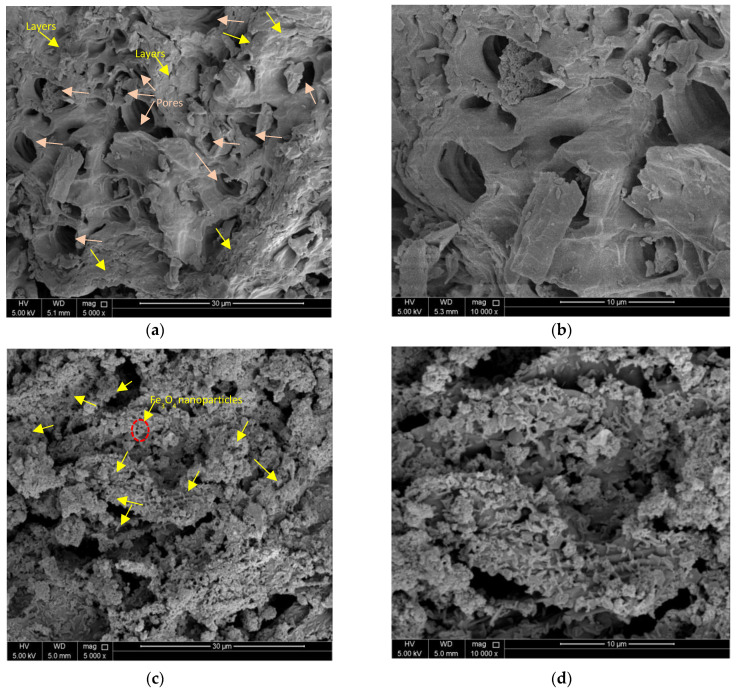

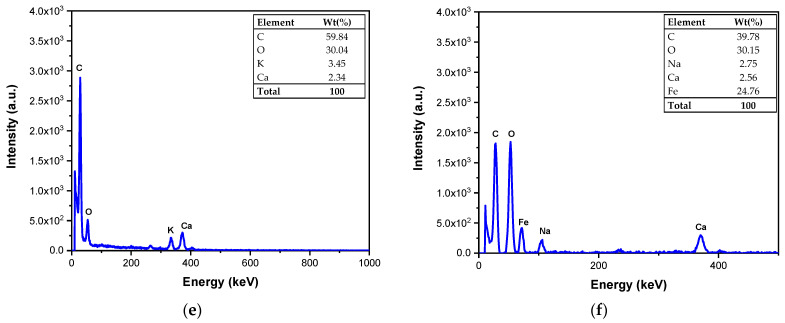
SEM micrographs of (**a**,**b**) OPBC, (**c**,**d**) MAG-OBPC at 5000× and 10,000× magnifications, (**e**,**f**) EDX analyses of OPBC and MAG-OPBC, respectively.

SEM and TEM analyses were executed to examine the surface morphology of Fe_3_O_4_ nanoparticles loaded biochar. Figure 5 displays the topographical study and the EDX charts of OPBC and MAG-OPBC. The SEM micrographs in Figure 5a,b depict the presence of many cavities and pores on the surface of the OPBC because of the thermal treatment of the peels. In contrast, the SEM micrograph for MAG-OPBC in Figure 5c,d shows the coverage of the surface of the OPBC with the magnetic nanoparticles, which have been proved by FT-IR, CHN, and Raman analyses. The EDX analysis of the OPBC sample in Figure 5e exposed a high concentration of carbon (59.84%) and oxygen (30.04%) in the presence of potassium (3.45%) and calcium (2.34%). On the other hand, the EDX analysis of MAG-OPBC, Figure 5f shows a lower concentration of carbon compared to OPBC (39.78%) due to the presence of magnetic nanoparticles on the surface of OPBC, an issue that was proven by the presence of a high concentration of iron (24.76%) and oxygen (30.15%). 

**Figure 6:**
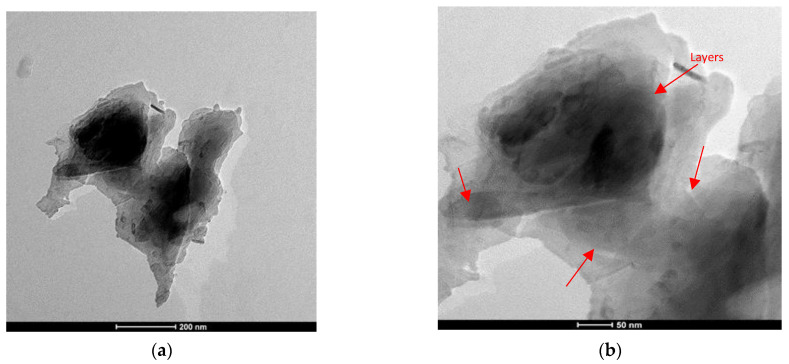

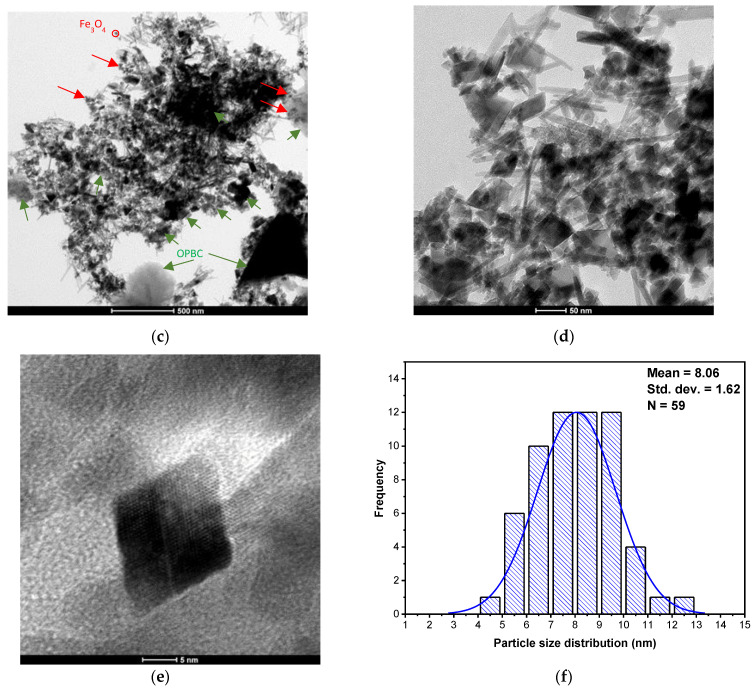
TEM images of OPBC at (**a**) 200 nm, (**b**) 50 nm, and MAG-OPBC at (**c**) 500 nm, (**d**) 50 nm, (**e**) 5 nm, and (**f**) Fe_3_O_4_ particle size distribution (PSD) for MAG-OPBC.

The microstructural characterization of the magnetic nanoparticles loaded onto the OPBC surface using TEM analysis is presented in Figure 6. The findings of the TEM analysis align well with the SEM micrographs. In Figure 6a,b, the OPBC surface appears smooth without any particles. On the other hand, the surface of the MAG-OPBC is rough, and Fe_3_O_4_ nanoparticles are easily visible, as shown in Figure 6c–e. Synthesized Fe_3_O_4_ nanoparticles had an average size of 8.06 ± 1.62 nm (Figure 6f) and small particle size distribution of 1.62 nm, proving the creation of uniformly sized Fe_3_O_4_ nanoparticles.

### 3.2. Adsorption Study

#### 3.2.1. Plackett–Burman Design (PBD)

The main objective of the present investigation is to set out an economical and green adsorbent by the reutilization of an abundantly available waste material, orange peels. Our target was also to enhance the adsorption capacity of the biochar-derived adsorbent, OPBC. This objective could be achieved by impregnation of the pristine candidate with magnetite nanoparticles (MAG-OPBC). Controlling and boosting the performance of either adsorbent could be achieved by controlling the process variables. Thus, an experimental design was instigated to initially screen the statistical significance of four variables affecting the removal of DNB. PBD was selected for this purpose. As a screening design, PBD is a simple design in which the number of experimental runs is the smallest multiple of four (˃the number of variables) and is used when only the main effects are concerned. Therefore, PBD is commonly used as a prelude to an optimization phase (coupled to the desirability function tool in this investigation) to ensure that all chosen variables do certainly and significantly contribute to the measured response, and hence further saving on the runs to be performed [55]. Moreover, PBD permits the determination of the main effects of all variables with the minimum variance with no concerns from the variable-variable interactions or the non-linear effects. Therefore, PBD is always of choice for robustness, ruggedness tests performed for the sake of method validation [43,44,55,56]. Compared to the most popular screening design, full factorial design, where a relatively large number of runs is needed to cover the main effects and all the possible combinations, PBD offers an economical solution where only the main effects are considered [55,56]. The design matrix, the obs. and pred. responses, together with the RE values, are shown in Table 2. As could be realized from the obtained data, the values of RE were apparently small enough to express the model’s accuracy.

#### 3.2.2. Quality Charts and Analysis of Variance (ANOVA)

**Figure 7:**
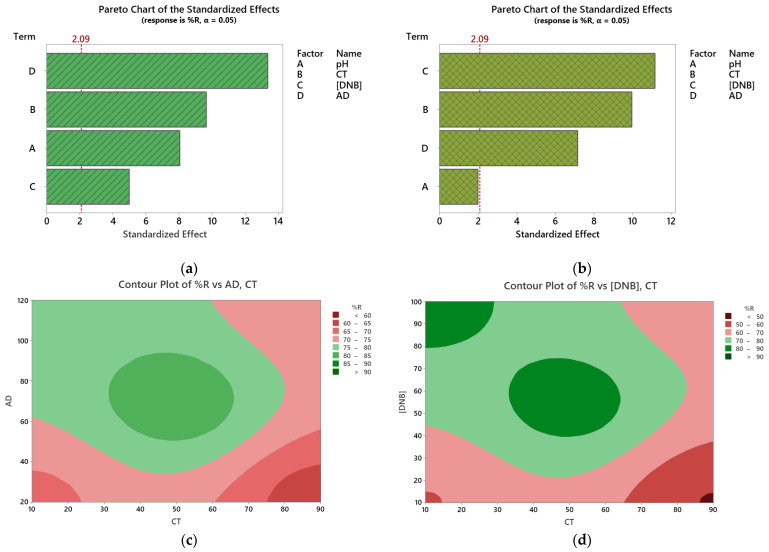

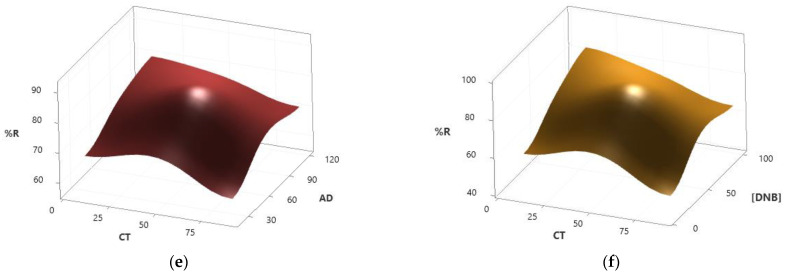
PBD analysis results: Pareto charts: (**a**) OPBC, (**b**) MAG-OPBC; Contour 2D-plots: (**c**) OPBC, (**d**) MAG-OPBC; and Surface 3D-plots: (**e**) OPBC, (**f**) MAG-OPBC.

The statistical impact of each variable (main effects) was assessed using Pareto chart. As shown in Figure 7a,b, variables surpassing the reference line are denoted as significant. Figure 7a shows that AD (D) was the variable that mainly affects the removal of DNB using OPBC, compared to [DNB] (C) in case of MAG-OPBC (Figure 7b). As also could be concluded from the Pareto chart, pH (A) was statistically insignificant in case of MAG-OPBC. In the same itinerary, similar conclusions could be derived from ANOVA testing operated at 95.0 confidence interval. Obtained results are displayed in Table S2. Variables with *p*-value ˃ 0.05 are statistically insignificant. For both adsorbents, the lack-of-fit was insignificant, suggesting the linearity of data. 

Figure 7c–f shows the contour and surface plots portraying design data in the 2D and 3D formats, correspondingly. For contour plots, the maroon and dark green regions denote areas with the lowest and highest %R of DNB, respectively. In the 3D plots, however, elevated ridges are the regions with the highest %R.

#### 3.2.3. Regression Equations

Regression models (main effects) are given by Equations (1) and (2) for OPBC and MAG-OPBC, respectively. As reflected by the regression models, the coefficient next to each variable signifies the magnitude of this variable, while the sign denotes the direction of the impact of this variable.

ln(%R_OPBC_) = 4.0734 + 0.01535 pH − 0.001352 CT + 0.000673 [DNB] + 0.001620 AD + 0.1793 Ct Pt, R^2^ = 96.75%, R^2^–adj = 95.72%, R^2^–pred = 94.08% (1)

ln(%R_MAG-OPBC_) = 3.9451 + 0.00763 pH − 0.002788 CT + 0.003003 [DNB] + 0.001735 AD + 0.3023 Ct Pt, R^2^ = 96.45%, R^2^–adj = 95.32%, R^2^–pred = 93.28% (2)

As per model summaries given following each regression model, both R^2^ and R^2^–adj were high enough, indicating model linearity. The R^2^-pred was used to assess the capacity of the proposed model to expect the response in case of any new observations, where the greater the value of R^2^–pred, the better the model’s predictive capability. Running the individual desirability function (*d*)—a statistical tool used to attest the capability of a certain factorial combination to maximize the response-delivered the data in Table 3. The closer the value of *d* to 1.0000, the more suitable the proposed factorial recipe. As shown in Table 3, maximum removal of DNB was attained using MAG-OPBC where %R = 98.51%

### 3.3. Equilibrium and Kinetic Studies for DNB Adsorption onto OPBC and MAG-OPBC

#### 3.3.1. Adsorption Isotherms

The adsorption of DNB onto OPBC and MAG-OPBC was studied using four models: Langmuir, Freundlich, Temkin, and Dubinin–Radushkevich (D–R) [57,58,59,60,61]. 

**Figure 8:**
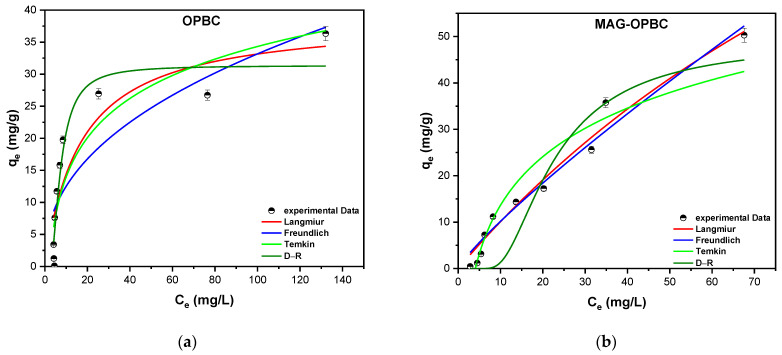
Adsorption equilibrium isotherms for the adsorption of DNB onto (**a**) OSBC and (**b**) MAG-OSBC sorbents.

According to the Langmuir model applied in this system, the following can be suggested: (I) All the adsorption sites on the adsorbent surface are equivalent with the same adsorption energy, (II) the adsorbed species occupy one location on the adsorbent surface without any interaction between them, and (III) the adsorption mostly takes place on the surface of the adsorbent. The Langmuir isotherm is represented by Equation (3) and Figure 8a,b, which illustrate the removal of DNB using OPBC and MAG-OPBC, respectively.

(3) $$q_{e}=\frac{q_{m}{\;K}_{L}{\;C}_{e}}{1+K_{L}{\;C}_{e}}$$

The equation features two parameters, *q_m_* and K_L_, which respectively stand for the maximum adsorption capacity and Langmuir equilibrium coefficient. Equation (4) also presents an alternative way of expressing this relationship in a dimensionless form as follows:

(4) $$R_{L}=\frac{1}{1+K_{L}\;C_{0}}$$

The desirability of adsorption can be evaluated by using the *R_L_* value, which is the DNB separation factor divided by the initial concentration (C_0_) measured in mg/L. The *R_L_* value is indicative of the type of adsorption. For both OPBC and MAG-OPBC, the *R_L_* value was less than 1, indicating that the process was favorable. As the concentration of DNB increased, the adsorption process became irreversible, and the maximum adsorption capacity (*q_m_*) for DNB was 38.75 mg/g and 172.43 mg/g for OPBC and MAG-OPBC, respectively. These results confirm the outcomes of the PBD. The data collected for the adsorption of DNB onto both OPBC and MAG-OPBC (Figure 8a,b) demonstrate that DNB adsorption onto MAG-OPBC matches well with the Langmuir isotherm, with R^2^ value of 0.9704. 

On the other hand, Equation (5) illustrates the Freundlich model:

(5) $$q_{e}=K_{F}C_{e}^{\frac{1}{n}}$$

The equation includes the Freundlich coefficients, which are denoted as *K_F_* (mole·g^−1^) (L·mole^−1^) and 1/*n*. These coefficients indicate the adsorbent capacity, the difference in adsorption intensity, and the deviation from linearity. Table 4 presents the calculated values of these parameters for the Freundlich isotherm. OPBC has a 1/*n* value of 0.42 and an *n* value of 2.38, while MAG-OPBC has a 1/*n* value of 0.86 and an *n* value of 1.16. Therefore, MAG-OPBC has a greater adsorption potential towards DNB drug than OPBC. 

Equation (6) represents the Temkin isotherm, which explains the interaction between the sorbate and the sorbent.

(6) $$q_{e}=\frac{RT}{b_{T}}\;\ln\left(A_{T}\;C_{e}\right)$$

The *A_T_* constant in this paradigm corresponds to the Temkin isotherm, while *R* refers to the universal gas constant (8.314 J/mol·K), *b_T_* denotes the Temkin isotherm constant, and *T* represents the temperature in Kelvin. Based on Figure 8a–d and Table 4, OPBC displays an adsorption energy of 280.09 J/mol, while MAG-OPBC exhibits an adsorption energy of 164.25 J/mol. These findings indicate that DNB is effectively adsorbed onto the surface of both OPBC and MAG-OPBC, which aligns with the results obtained from the Langmuir and Freundlich models. 

Equation (7) represents the Dubinin–Radushkevich (D–R) model, which was investigated to estimate the adsorption mechanisms of DNB onto OPBC and MAG-OPBC.

(7) $$q_{e}=q_{s}\cdot exp(\beta \cdot \;\epsilon^{2})$$

Equation (8) uses *β*, the activity coefficient, to calculate the DNB sorption energy, *E* (kJ/mol), where *q_s_* denotes the saturation capacity of both OPBC and MAG-OPBC. The Polanyi potential is determined by *ϵ*, which can be calculated using Equation (8). Similarly, the value of *E* can be obtained using Equation (9).

(8) $$\epsilon =RT\left(1+\frac{1}{C_{e}}\right)$$

(9) E=12β

The results obtained for both adsorbents suggest that the DNB adsorption energy onto OPBC is 5.85 kJ/mol, whereas the MAG-OPBC adsorption energy is 1.97 kJ/mol. These values indicate that the adsorption of DNB onto both OPBC and MAG-OPBC is physisorption, with an adsorption energy of less than 7 kJ/mol additionally, the surface area of the nanosorbent appears to have a significant impact on the adsorption process. The adsorption of DNB onto OPBC is well-fitted with the D–R isotherm, as evidenced by an R^2^ value of 0.9281.

#### 3.3.2. Kinetic Models

**Figure 9:**
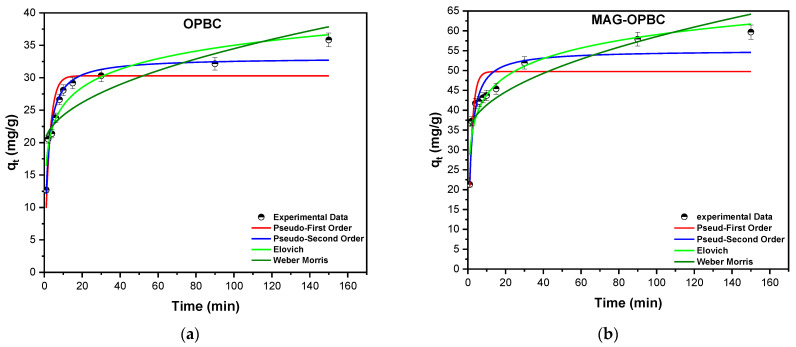
Kinetic studies for the sorption of DNB onto (**a**) OPBC and (**b**) MAG-OPBC adsorbents.

To investigate the adsorption of DNB onto OPBC and MAG-OPBC, four kinetic models were employed: Pseudo-first-order (PFO), pseudo-second-order (PSO), Elovich, and Weber–Morris (WM) [62,63,64,65] (Figure 9a,b). Table 5 provides the estimated parameters for each model. The results indicate that the PSO model provides a good fit for the adsorption of DNB onto both OPBC and MAG-OPBC, with R^2^ values of 0.9353 and 0.8782, respectively. These findings suggest that the rate of the adsorption interaction between DNB and the two nanosorbent is controlled by the drug and adsorbent concentrations, as demonstrated by Equation (10):

(10) $$OPBC\;\&\;MAG-OPBC+DNB\;\left(\overset{k}{\to }\;\right)\;\left\{DNB–OPBC\right\}\;or\;\left\{DNB–MAG-OPBC\right\}$$

Using the Elovich model, the initial adsorption rate for DNB was determined to be 237.04 mg·g^−1^·min^−1^ for OPBC, which is lower than the initial adsorption rate of MAG-OPBC (513.72 mg·g^−1^·min^−1^). However, the R^2^ value of the Weber–Morris (WM) model was too low for both adsorbents, and thus, it was unable to provide an adequate explanation of the adsorption of DNB onto the OPBC and MAG-OPBC nanosorbent in comparison to the other models.

### 3.4. Proposed Adsorption Mechanism

**Figure 10:**
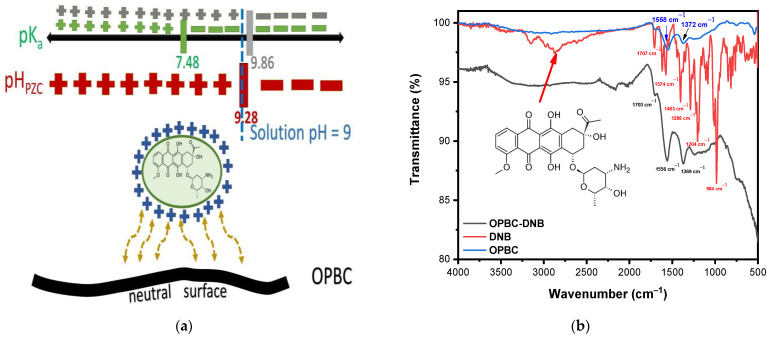

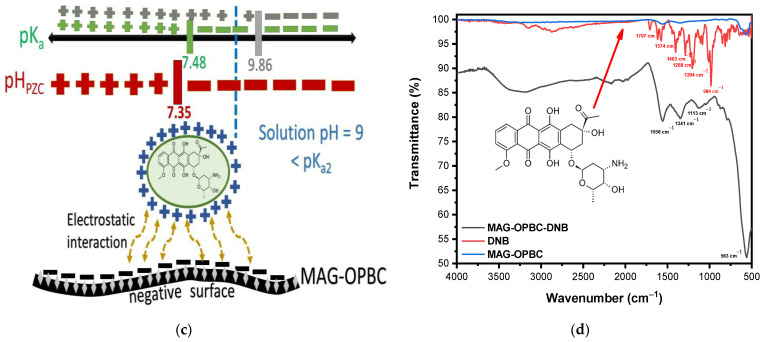
(**a**) Proposed mechanism for adsorption of DNB onto OPBC, (**b**) FT-IR spectrum of OPBC before and after adsorption of DNB, (**c**) proposed mechanism for adsorption of DNB onto MAG-OPBC, (**b**,**d**) FT-IR spectrum of MAG-OPBC before and after adsorption of DNB.

Electrostatic interactions, hydrogen bonding, intra-particle interactions, and surface diffusions might all be used to explain how DNB adheres to both OPBC and MAG-OPBC, Figure 10a–d.

1. Electrostatic interactions occur when the DNB-charged functional groups interact with the charged surface of either OPBC or MAG-OPBC. The pH may impact these interactions since it can modify the surface charge of both the DNB and the as-prepared adsorbent [66,67]. As shown in Figure 10a,c, the optimum pH greatly affects the surface of both the adsorbate and the adsorbent. For MAG-OPBC, the optimum pH was found to be 9.0, which is less than the pK_a2_ of DNB but higher than the pH_pzc_ for MAG-OPBC. This results in a positive charge on the surface of the drug molecules and a negative charge on the surface of MAG-OPBC, leading to a strong electrostatic interaction between DNB and MAG-OPBC and increasing the removal efficiency. On the other hand, the data obtained for OPBC show that the optimum pH is also 9.0, and the pH_pzc_ of OPBC is also 9.28. This results in a neutral surface charge, which negatively affects the electrostatic interaction and thus, the removal efficiency of OPBC.
2. Chemical bonding can occur between DNB and both OPBC and MAG-OPBC surfaces through covalent or ionic bonds. This mechanism is significant for DNB, which has functional groups that can participate in chemical reactions with the surface of the nanosorbent. Different functional groups with the adsorbate’s benzene or other aromatic ring structures could have electron-rich or electron-deficient groups that facilitate the interaction (Figure 10b,d). Additionally, there may be hydrogen-bonding interactions between the oxygen atoms (H-acceptors) in the nanosorbent and the hydrogen of the hydroxyl groups (H-donors) on the DNB drug. This type of interaction is commonly referred to as hydrogen bonding between dipoles.
3. Intra-particle and surface diffusions mechanism: Intra-particle diffusion refers to the movement of adsorbate molecules within the pores of the adsorbent particle [68,69]. In the case of OPBC and MAG-OPBC, many pores existed on the surface, as confirmed by the SEM and BET analyses, and the adsorbate (DNB) diffuses into the pores of the adsorbent and interacts with the surface of the nanosorbent. Surface diffusion, conversely, refers to the movement of adsorbate molecules on the surface of the OPBC and MAG-OPBC adsorbent. In the case of OPBC, the DNB molecules diffuse along the surface of the biochar and interact with either the surface functional groups or the magnetic nanoparticles of the MAG-OPBC. Both mechanisms are important for the description of adsorption of DNB onto OPBC and MAG-OPBC because they explain the increased interaction between DNB and the adsorbent surface, which enhances the adsorption process.

## 4. Conclusions

This study aimed to remove DNB from synthetic wastewater using two adsorbents produced from orange peels, a copiously abundant agro-waste. The performances of the as-prepared biochars (OPBC) and magnetite-impregnated candidate (MAG-OPBC) were compared, and MAG-OPBC was found to be more effective. Different characterization techniques were used to understand the adsorbent behavior, including TGA/*d*TA, FT-IR, point-of-zero-charge, BET, SEM, and TEM analyses. To ensure sustainability and method greenness, eco-structuring of the prepared adsorbent was approached by controlling the variables impacting the adsorption process using the PBD. The Pareto chart confirmed that the most significant variables were the adsorbent dose. In the case of pristine biochar and the [DNB] in the case of MAG-OPBC. The desirability function was used to determine the variables that maximize the removal of DNB. The highest removal efficiency of DNB was 86.46% and 98.51% for OPBC and MAG-OPBC, respectively. The optimum adsorption conditions were proposed by the desirability function to be pH = 9.0, CT = 10 min, [DNB] = 100 ppm, and AD = 120 mg/13 mL. Nonlinear equilibrium studies revealed that DNB adsorption onto MAG-OPBC fits well with the Langmuir isotherm, compared to the D–R isotherm model in the case of OPBC. The maximum adsorption capacity was found to be 38.75 and 172.43 mg/g for OPBC and MAG-OPBC, respectively. The D–R isotherm showed that the adsorption of DNB onto both adsorbents follows a physisorption mechanism with an adsorption energy of ˂7 kJ/mol. The Elovich model showed that the initial adsorption rate for DNB using OPBC was lower than that of MAG-OPBC. The PSO model was able to explain the adsorption kinetics of DNB onto both adsorbents. The adsorption of DNB onto the two adsorbents can be described using several adsorption mechanisms, including electrostatic interactions, intra-particle interactions, and surface diffusions.

## Figures and Tables

**Table 1:** Removal of antineoplastics from contaminated waters using different strategies.

Antineoplastic Drug	Strategy	Source of Drug	Process Optimization	Removal Efficiency	Ref.
Daunorubicin	Adsorption using two sorbents: Orange peels’ biochar (OPBC) and the magnetic counterpart (MAG-OPBC)	Synthetic wastewater	Plackett–Burman Design, PBD	OPBC: 86.46% MAG-OPBC: 98.51%	This Study
Daunorubicin, doxorubicin	Adsorption on synthesized hydrophilic silica aerogels	Synthetic pharmaceutical wastewater	Univariate analysis	Under the given working conditions of 60 ppm [drug], a contact time of 60 min, 0.15 g of adsorbent, a wastewater volume of 25 mL, and at ambient temperature, the %R of doxorubicin was 97.11%, which was only slightly higher compared to daunorubicin at 96.89%.	[7]
Bleomycin and vincristine	Biosorption on fungal biomass	Aqueous solution	Central composite design (CCD)	Dead biomass showed a maximum removal of 38% ± 5% in the case of bleomycin, compared to 20% ± 6% using the active biomass in the case of vincristine	[8]
Cyclophosphamide, azathioprine, methotrexate, doxorubicin, epirubicin, flutamide, mitotane, and tamoxifen	Anaerobic osmotic membrane bioreactor	Synthetic domestic wastewater	Univariate analysis	Adsorption removed doxorubicin, epirubicin, and tamoxifen, while biodegradation and forward osmosis rejection removed methotrexate and cyclophosphamide, respectively. Mitotane, azathioprine, and flutamide were eradicated through a combination of biodegradation and adsorption.	[9]
Flutamide	Biosorption using living and dead biomass of *Chlorella vulgaris*	Synthetic wastewater	RSM	The living microalga exhibited a higher maximum sorption capacity of 26.8 mg g^−1^ compared to the dead biomass at 12.5 mg g^−1^. By utilizing the living biomass, a %R of 98.5% was attained.	[10]
Flutamide	CoFe_2_O_4_ nanoparticles in Fenton/ozone oxidation process	Synthetic wastewater	RSM	The drug removal efficiency reached 67.7% under optimal conditions.	[11]
Cyclophosphamide (CP) and its metabolite: 4-ketocyclophosphamide	A pair of membrane bioreactors were operated in parallel, where one was fed with the drug and its primary metabolites, while the other received no drug.	Municipal wastewater	Univariate analysis	The operational conditions studied resulted in an 80% removal of both CP and its metabolite, 4-ketocyclophosphamide.	[12]
Flutamide	A treatment process that combines photocatalysis and algae in a single stage.	Synthetic wastewater	Response surface methodology (RSM)	The drug %R of the individual algae treatment and individual photocatalysis were 67.2% and 66.6%, respectively, under the optimum conditions. However, the combined process achieved a significantly higher removal efficiency of 85.1%.	[13]
Paclitaxel, etoposide, cyclophosphamide, ifosfamide	Nanofiltration using Desal 5DK and NF270 membranes	laboratory degree water, synthetic urine, actual secondary effluent	No investigation of variables was made	Compared to the NF270 membrane, the Desal 5DK membrane demonstrated greater effectiveness. The Desal 5DK membrane exhibited an average rejection rate of more than 89% for the four drugs that were introduced into both synthetic urine and real secondary effluent.	[14]

**Table 2:** Studied variables, matrix, as well as the observed (Obs.) and the predicted (Pred.) for the PBD executed to investigate the statistical significance of each variable. Values of Obs. and Pred. responses are contrasted using the value of the relative error (RE).

Variable					Low (−)		Central (0)		High (+)	
pH (A, pH unit)					3.0		6.0		9.0	
Contact Time (CT, B, min)					10.0		50.0		90.0	
Initial DNB Concentration ([DNB], C, ppm)					10.0		55.0		100.0	
Sorbent Dosage (AD, D, mg/13 mL)					20.0		70.0		120.0	
**Trial Number**	**Variable–Coded Levels**				* %R_OPBC_			* %R_MAG-OPBC_		
	**A**	**B**	**C**	**D**	**Obs.**	**Pred.**	**RE**	**Obs.**	**Pred.**	**RE**
01	−	+	+	−	56.87	60.19	0.06	57.44	57.43	0.00
02	+	+	+	−	62.80	65.99	0.05	62.88	60.22	0.04
03	−	−	+	+	76.02	78.86	0.04	96.30	85.51	0.13
04	+	−	+	−	70.62	73.53	0.04	76.15	75.26	0.01
05	+	+	+	+	75.46	77.59	0.03	78.84	71.62	0.10
06	0	0	0	0	83.37	83.73	0.00	86.61	84.82	0.02
07	0	0	0	0	82.62	83.73	0.01	85.58	84.82	0.09
08	+	−	+	+	84.04	86.46	0.03	86.25	89.51	0.04
09	−	−	−	+	71.22	74.22	0.04	63.51	65.26	0.03
10	+	+	−	+	69.68	73.04	0.05	57.95	54.66	0.06
11	+	−	−	+	75.02	81.38	0.08	65.45	68.32	0.04
12	−	+	+	+	67.74	70.77	0.04	68.80	68.42	0.01
13	0	0	0	0	83.39	83.73	0.00	87.49	84.82	0.03
14	−	+	−	+	70.04	66.62	0.05	54.31	52.21	0.04
15	+	+	−	−	64.91	62.11	0.05	43.06	45.96	0.06
16	−	−	−	−	65.45	63.12	0.04	55.03	54.86	0.00
17	−	−	+	−	70.05	67.06	0.04	70.06	71.89	0.03
18	+	−	+	+	91.76	86.46	0.06	85.57	89.51	0.04
19	+	+	−	−	65.03	62.11	0.05	45.03	45.96	0.02
20	0	0	0	0	86.04	83.73	0.03	82.41	84.82	0.03
21	−	+	+	−	62.27	60.19	0.03	53.21	57.52	0.07
22	0	0	0	0	81.02	83.73	0.03	81.18	84.82	0.04
23	−	+	−	+	70.08	66.61	0.05	48.30	52.21	0.07
24	0	0	0	0	86.05	83.73	0.03	85.80	84.82	0.01
25	−	−	−	−	65.47	63.12	0.04	54.83	54.83	0.00
26	+	−	−	−	72.93	69.21	0.05	60.08	57.47	0.05

* The response, %R, was computed using the equation: $\left(\%R\right)=\frac{C_{0}-C_{e}}{C_{0}}\;\times \;100\%$, where C_0_ and C_e_ denote the initial and equilibrium concentrations of DNB.

**Table 3:** Optimum conditions and desirability function *d*-values.

Adsorbent	Optimum Conditions	Desirability Function (*d*)
OPBC	pH = 9.0, CT = 10 min, [DNB] = 100 ppm, AD = 120 mg/13 mL	*d* = 0.8481, %R = 86.46%
MAG-OPBC	pH = 9.0, CT = 10 min, [DNB] = 100 ppm, AD = 120 mg/13 mL	*d* = 0.8275, %R = 98.51%

**Table 4:** Calculated parameters for the adsorption equilibrium models used for the adsorption of both DNB onto OPBC and MAG-OPBC.

Langmuir	*q_m_* (mg/g)	K_L_ (L·mole^−1^)	R^2^	
OPBC	38.75	0.059	0.8267	
MAG-OPBC	172.43	0.006	0.9704	
**Freundlich**	**1/*n***	***K_F_* (mole/g) (L/mole)^1/n^**	**R^2^**	
OPBC	0.42	4.721	0.7547	
MAG-OPBC	0.86	1.416	0.9639	
**Temkin**	***b_T_* (J/mole)**	***A_T_* (L/mole)**	**R^2^**	
OPBC	280.09	0.484	0.8434	
MAG-OPBC	164.25	0.247	0.9150	
**D–R**	β	***E* (kJ/mole)**	***q_m_* (mg/g)**	**R^2^**
OPBC	1.46 × 10^−8^	5.85	31.34	0.9281
MAG-OPBC	1.29 × 10^−7^	1.97	48.94	0.8609

**Table 5:** Calculated data for four kinetic models used for the adsorption of DNB onto OPBC and MAG-OPBC.

Models	Parameter	Value	
		OPBC	MAG-OPBC
Pseudo-first order (PFO) $\frac{dq_{t}}{dt}$*= k*_1_(*q_e_ − q_t_*)	*K*_1_ (min^−1^)	0.397	0.541
	*q_e_* (mg/g)	30.29	49.74
	R^2^	0.78114	0.7074
Pseudo-second order (PSO) $\frac{dq_{t}}{dt}$ *= k*_2_(*q_e_ − q_t_*)^2^	*K*_2_ (g·mg^−1^·min^−1^)	0.017	0.012
	*q_e_* (mg/g)	33.09	55.11
	R^2^	0.9353	0.8782
Elovich model *q_t_=* $\frac{1}{\beta }\;\times ln\left(1+\alpha \beta t\right)$	α	237.04	513.72
	β	0.248	0.152
	R^2^	0.9130	0.8506
Weber−Morris model (WM) $q_{t}=K_{I}t^{0.5}+C$	*K_I_*	1.50	2.518
	*C*	19.46	33.36
	R^2^	0.6926	0.7268

## Data Availability

The data presented in this study are available within this article. Further inquiries could be directed to the authors.

## Supplementary Materials

The following supporting information can be downloaded at: https://www.mdpi.com/article/10.3390/nano13091444/s1, Table S1: BET analysis of the two sorbents; OPBC and MAG-OPBC; Table S2: ANOVA findings using OPBC and MAG-OPBC.
