# Supplementary material for: Performance of Pristine versus Magnetized Orange Peels Biochar Adapted to Adsorptive Removal of Daunorubicin: Eco-Structuring, Kinetics and Equilibrium Studies
Source: Nanomaterials (Basel). 2023 Apr 23;13(9):1444. doi: 10.3390/nano13091444 (PMC10179814; doi:10.3390/nano13091444)
Supplement: Supplementary file 1 [file nanomaterials-13-01444-s001.zip › nanomaterials-2348455-supplementary.pdf]

Table S1. BET analysis of the two sorbents: OPBC and MAG-OPBC.

Parameters	OPBC	MAG-OPBC
Langmuir surface area (m^2/g)	6.99	60.76
Total pore volume (cm^3/g)	0.021	0.263
Average pore radius (Å)	110.5	84.4

Table S2. ANOVA findings using OPBC and MAG-OPBC.

		OPBC				MAG-OPBC			
**Source**	**DF**	**Adj SS**	**Adj MS**	**F-Value**	**P-Value**	**Adj SS**	**Adj MS**	**F-Value**	**P-Value**
Model	6	0.355030	0.059172	94.29	0.000	1.29146	0.215244	85.96	0.000
Blocks	1	0.025076	0.025076	39.96	0.000	0.00912	0.009117	3.64	0.072
Linear	4	0.206087	0.051522	82.10	0.000	0.64078	0.160195	63.97	0.000
pH	1	0.040670	0.040670	64.81	0.000	0.01006	0.010063	4.02	0.059
CT	1	0.058515	0.058515	93.24	0.000	0.24865	0.248648	99.30	0.000
[DNB]	1	0.015678	0.015678	24.98	0.000	0.31207	0.312073	124.63	0.000
AD	1	0.112143	0.112143	178.70	0.000	0.12855	0.128554	51.34	0.000
Curvature	1	0.148343	0.148343	236.38	0.000	0.42188	0.421879	168.48	0.000
Error	19	0.011923	0.000628			0.04758	0.002504		
Lack-of-Fit	12	0.009448	0.000787	2.23	0.147	0.03782	0.003152	2.26	0.143
Pure Error	7	0.002475	0.000354			0.00976	0.001394		
Total	25	0.366954				1.33904	0.215244	85.96	0.000
